# Orthogonal cation and anion template-directed synthesis of a heteroditopic [3]catenane for ion-pair recognition

**DOI:** 10.1039/d5sc05942a

**Published:** 2026-04-02

**Authors:** Hui Min Tay, Andrew Docker, Antonio J. Martínez-Martínez, Paul D. Beer

**Affiliations:** a Chemistry Research Laboratory, Department of Chemistry, University of Oxford Mansfield Road Oxford OX1 3TA UK paul.beer@chem.ox.ac.uk; b Yusuf Hamied Department of Chemistry, University of Cambridge Lensfield Road Cambridge CB2 1EW UK; c Department of Chemistry and Center for Research in Sustainable Chemistry (CIQSO), University of Huelva, Campus El Carmen 21007 Huelva Spain

## Abstract

The first orthogonal template strategy for the synthesis of higher order interlocked structures is described. Orthogonal cation and anion templates were employed in a stepwise fashion to construct a hetero[3]catenane composed of structurally distinct macrocyclic components. Importantly, the [3]catenane contains two interlocked binding cavities tailored for cation and anion binding, enabling it to function as a heteroditopic ion-pair receptor. The co-conformational dynamics and cation/anion binding properties of the [3]catenane were investigated by ^1^H NMR studies, which displayed temperature-dependent and guest-induced changes in chemical shift and linewidth. The potent ion-pair binding capability of the [3]catenane was confirmed by single-crystal X-ray diffraction studies, which showed the [3]catenane to recognise a sodium chloride ion-pair in a mechanically bonded receptor-separated ion-pair binding mode, wherein the two guest ions are encapsulated within their respective interlocked host cavities.

## Abstract 2

A hetero[3]catenane was synthesised using an orthogonal cation and anion templating strategy. The interlocked structure contains binding sites that cooperatively recognise a NaCl ion-pair, demonstrating potential for higher-order heteroditopic hosts.

## Graphical abstract

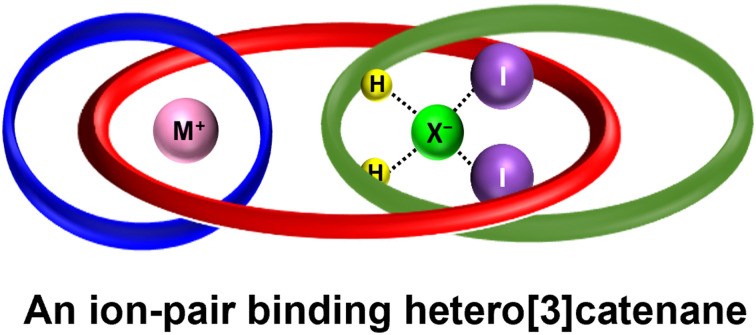

## Introduction

Since the first report of a statistical catenane synthesis by Wassermann more than 60 years ago,^1^ the topological complexity of this class of molecules has continued to both fascinate and challenge supramolecular chemists. Despite the growing arsenal of template-directed methods to facilitate mechanical bond formation, thus far only the simplest ‘Hopf’ link, a [2]catenane with two molecular crossing points, can be accessed in a straightforward manner. In contrast, the preparation of higher order [*n*]catenanes (*n* ≥ 3) is complicated by additional considerations such as an increase in the number of bond-forming reactions required, competing equilibria leading to the formation of side products, and consequentially greater challenges associated with purification of the target [*n*]catenane. Another important consideration is the heightened possibility of topological isomerism as the number of macrocyclic components increases. As such, strategies for the preparation of higher-order catenanes have thus far been relatively limited,^2–20^ the majority of which utilise a single type of interaction (*e.g.* charge transfer interactions,^21–24^ metal-ligand coordination,^25–27^ hydrogen bonding^3,28^*etc.*). This typically gives rise to catenanes in which at least two of the macrocycles are identical, thus limiting the structural diversity of the interlocked products and consequentially restricting functionality.

The difficulty associated with preparing higher order catenanes has thus far limited their applications as sensors, switches and molecular machines. Despite this, their potential superiority over simpler interlocked systems has been highlighted in recent seminal reports. For example, Leigh and co-workers demonstrated unidirectional motion in a [3]catenane-based molecular motor, but not the [2]catenane analogue.^3^ Additionally, in the context of supramolecular host-guest chemistry, particularly ion-pair recognition, the use of higher order catenane receptors containing interlocked binding sites for both guest ions is a potential means to enhance the affinity and selectivity of ion-pair binding *via* a mechanical bond effect (MBE).^29^ The development of synthetic strategies to access these exotic molecules is therefore highly desirable and a promising avenue through which to extend the suite of applications for mechanically bonded architectures.

**Fig. 1:**
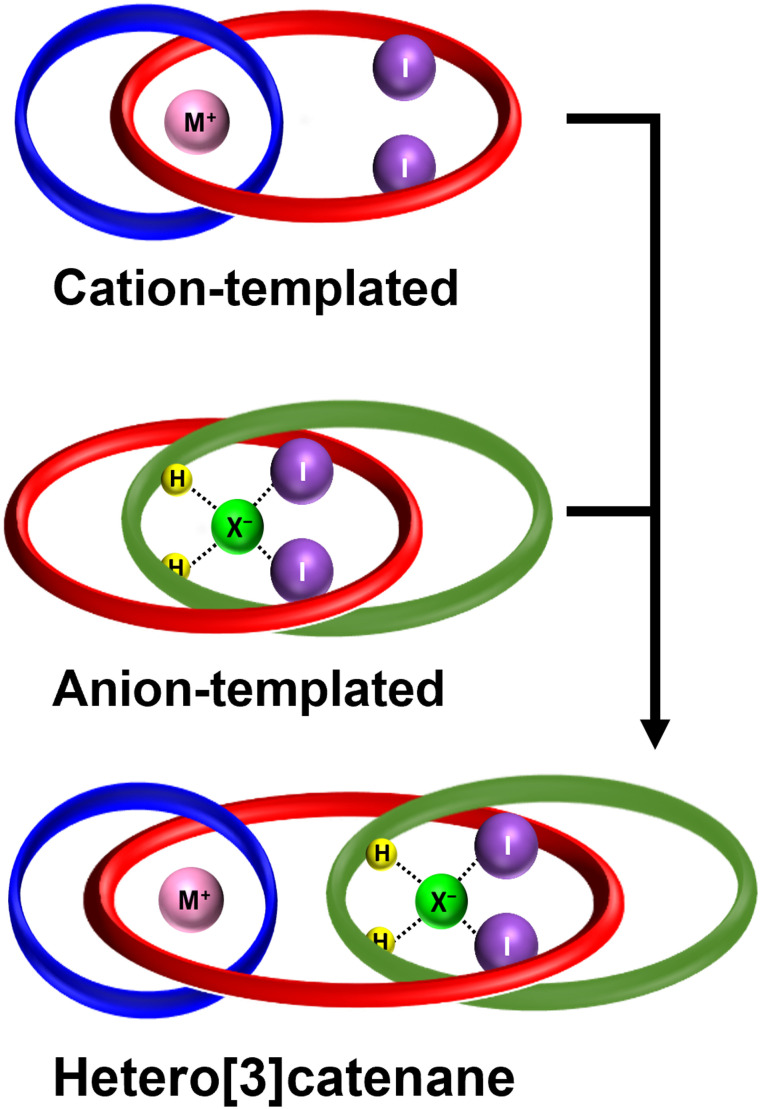
Cartoon representation of a heteroditopic[3]catenane for ion-pair recognition prepared *via* orthogonal cation and anion template-directed strategies.

We previously reported the cation-templated synthesis of a class of heteroditopic [2]catenanes and [2]rotaxanes containing a 3D poly(ethylene glycol)-based interlocked binding cavity for alkali metal cation recognition.^30–32^ We separately developed a chloride anion-template directed strategy for the synthesis of halogen bonding interlocked hosts capable of potent halide binding in aqueous-containing solvent mixtures *via* encapsulation of the anion guest in a binding cavity decorated with halogen bonding (XB) and hydrogen bonding (HB) donor groups.^33–35^ In both cases, the presence of pre-organised 3D binding cavities was demonstrated to significantly enhance both the binding affinities and selectivity profiles of the interlocked hosts for individual cation or anion guests *via* a MBE.^29^ We therefore envisaged that a heteroditopic [3]catenane containing interlocked binding cavities for both a cationic and anionic guest would have the potential to exhibit exceptional mechanical bond enhanced ion-pair binding capabilities^36^ (Fig. 1).

Herein, we report the sequential use of orthogonal alkali metal cation and chloride anion template-directed methodologies for the stepwise preparation of a heteroditopic [3]catenane 1, containing interlocked binding cavities complementary to both a cation and anion guest. Variable temperature ^1^H NMR studies provide insights into the dynamics of the interlocked macrocycles in the absence and presence of ion guest species. Additionally, solid state X-ray crystallographic investigations reveal the [3]catenane binding to a sodium chloride ion-pair in a host-separated binding mode, in which both ions are encapsulated in their respective interlocked binding pockets. Importantly, this represents, to the best of our knowledge, the first instance of using oppositely charged ions as orthogonal templates for the preparation of a higher-order [3]catenane capable of ion-pair binding, and serves to advance the development of novel strategies toward the synthesis of topologically complex and functionally potent host molecules.

## Results and discussion

### Host design and synthesis

**Scheme 1:**
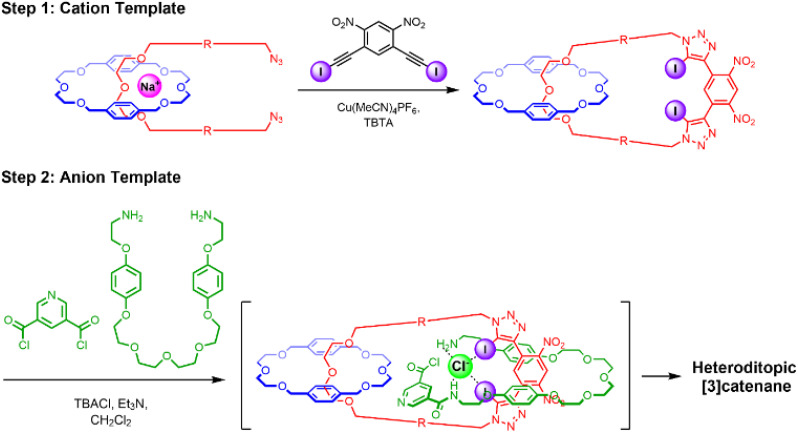
Proposed stepwise synthesis of a hetero[3]catenane *via* cation followed by anion template-directed methods.

**Scheme 2:**
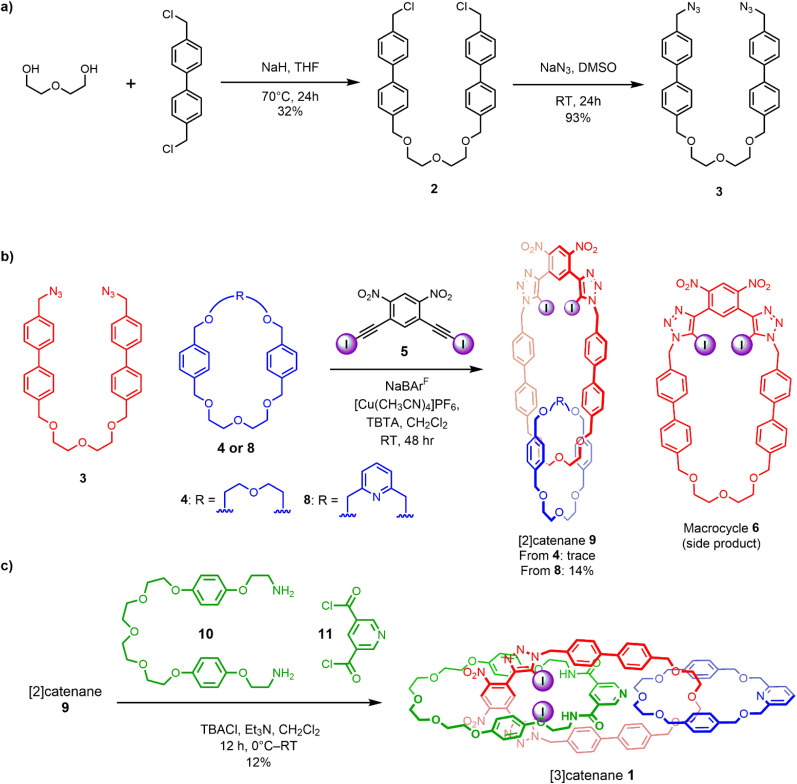
Synthesis of: (a) extended bis-azide precursor 3, (b) intermediate [2]catenane 9, (c) [3]catenane 1*via* orthogonal cation and anion template-directed methods.

Motivated by the efficacy of Chiu’s alkali metal cation^37,38^ and our own chloride anion^33^ templated-directed strategies for the preparation of interlocked host molecules for ion recognition, we sought to combine these orthogonal templating strategies to generate a hetero[3]catenane containing an oligo(ethylene glycol)-based interlocked cavity for cation binding alongside a XB- and HB-decorated cavity for halide binding. The proposed general synthetic approach involves two mechanical bond forming steps. Firstly, an alkali metal cation directs the formation of a [2]catenane, wherein, crucially, the CuAAC-mediated macrocycle clipping reaction generates a 4,6-dinitro-1,3-bis-iodotriazole group. This potent XB donor motif is then well-placed to facilitate a subsequent chloride template-directed amide condensation reaction between a bis-amine and a bis-acid chloride to form the third interlocked macrocycle (Scheme 1). Our previously reported [2]catenanes utilise an XB macrocycle containing a *p*-xylene based linker connecting the oligo-(ethylene glycol) cation binding site and the bis(iodotriazole) XB donor (denoted R in Scheme 1). However, the need for an expanded central macrocycle with a sufficiently large cavity to accommodate two threaded species necessitated the preparation of a longer bis-azide precursor. We therefore prepared a bis-azide in which biphenyl linkers separate the oligo(ethylene glycol) cation binding site from the terminal azide groups. Alkylation of di(ethylene glycol) with excess 4,4′-bis(chloromethyl)-1,1′-biphenyl in the presence of NaH produced dichloride 2 in 32% yield. Treatment of 2 with NaN_3_ in DMSO solution afforded the target bis-azide 3 in 93% yield (Scheme 2a). With the bis-azide in hand, the formation of a hetero[2]catenane *via* an alkali metal template-directed methodology was attempted (Scheme 2b). To this end, bis-azide 3, sodium tetrakis[3,5-bis(trifluoromethyl)phenyl]borate (NaBAr^F^_4_) and bis-di(ethylene glycol)-functionalised macrocycle 4 were pre-complexed in dry CH_2_Cl_2_, followed by dropwise addition of CH_2_Cl_2_ solutions containing 4,6-dinitro-1,3-bis-iodoalkyne 5 and [Cu(MeCN)_4_]PF_6_/TBTA. The reaction was allowed to stir for 3 days, after which TLC analysis indicated complete consumption of the bis-azide and the formation of a complex mixture of compounds, including the non-interlocked XB macrocycle as well as several more polar species. ESI-MS analysis of the crude mixture suggested that a trace amount of the desired [2]catenane had been formed, as indicated by the appearance of a small peak with *m*/*z* = 1433. The reaction was subjected to a basic work-up with aqueous EDTA solution and purification by preparative TLC. Unfortunately, however, the low yields and intractable side-products resulted in the desired [2]catenane not being isolated. The major side-product of the reaction appeared to be the non-interlocked XB macrocycle 6, which was isolated in 25% yield. Mass spectrometric analysis of the polar components of the mixture, which were formed in minute quantities, suggested the presence of a [2 + 2] macrocycle, as well as several oligomeric species. The failure to obtain the desired [2]catenane was attributed to the susceptibility of the 4,6-dinitro-1,3-bis-iodoalkyne towards de-iodination *via* nucleophilic attack, which when combined with the inherently low yield of hetero[2]catenane formation using macrocycle 4, likely generated only trace amounts of the desired catenane product.

These observations were concordant with a recent study by Chiu and co-workers,^37^ who noted that attempts to prepare hetero[2]catenanes *via* Na^+^ template-directed threading of acyclic di(ethylene glycol) precursors through the cavity of bis-di(ethylene glycol)-incorporated macrocycles, such as 4, typically resulted in low reaction yields. This was attributed to the poor directionality of metal cation binding of macrocycle-incorporated di(ethylene glycol) chains, leading to both desired internal threading and undesired external binding. The authors showed that hetero[2]catenanes yields could be improved through the use of 2,6-dimethanolpyridine-containing macrocycles, where sodium cation binding within the macrocycle cavity favoured the formation of a threaded pseudo[2]rotaxane species.^37^

These findings prompted us to investigate sodium ion template-directed hetero[2]catenane formation using a 2,6-dimethanolpyridine-containing macrocycle 8, which was prepared using modified literature procedures.^38^ Hetero[2]catenane formation was undertaken utilising macrocycle 8 and bis-azide 3 in the presence of a Na^+^ template (Scheme 2b). Pleasingly, the target hetero[2]catenane 9 was isolated in 14% yield following aqueous/EDTA work-up and purification by preparative TLC.

Evidence for the interlocked nature of 9 was obtained by comparing the XB [2]catenane's ^1^H NMR spectrum to that of the constituent macrocycles 6 and 8 (Fig. S8). Significant upfield perturbations were observed in the proton signals arising from macrocycle 8, presumably due to their enforced proximity to the shielding ring currents generated by the biphenyl spacers of macrocycle 6. In macrocycle 6, the most prominent changes were observed in the proton signals proximal to the ethylene glycol region, particularly H_i,j_ and H_h_ which moved upfield, suggesting that this region of the macrocycle participates in intercomponent interactions with 8. In contrast, only small perturbations occurred in the dinitrobenzene proton signals H_a_ and H_b_, which suggests that the XB motif of 6 remains sterically accessible for chloride binding. With the XB [2]catenane 9 in hand, the chloride template-directed preparation of a [3]catenane was undertaken (Scheme 2c). A solution of [2]catenane 9, bis-amine 10, Et_3_N and a source of the chloride templating agent in the form of its tetrabutylammonium salt (TBACl) in anhydrous CH_2_Cl_2_ was cooled to 0 °C, followed by dropwise addition of a solution of freshly prepared bis-acid chloride 11 in CH_2_Cl_2_. The reaction mixture was allowed to warm to room temperature and stirred overnight. ESI-MS analysis of the crude reaction mixture suggested the formation of the target [3]catenane, as evidenced by the appearance of an ion peak with *m*/*z* = 2062. Following aqueous work-up with K_2_CO_3_ solution and preparative TLC purification of the reaction mixture, a modest quantity of the putative [3]catenane 1 was isolated as an orange solid in approximately 12% yield, which was characterised by ^1^H NMR, mass spectrometry and solid state X-ray crystallography. In addition, the HB donor amide macrocycle 12 was also isolated for comparative purposes.

### ^1^H NMR and mass spectrometric studies

**Fig. 2:**
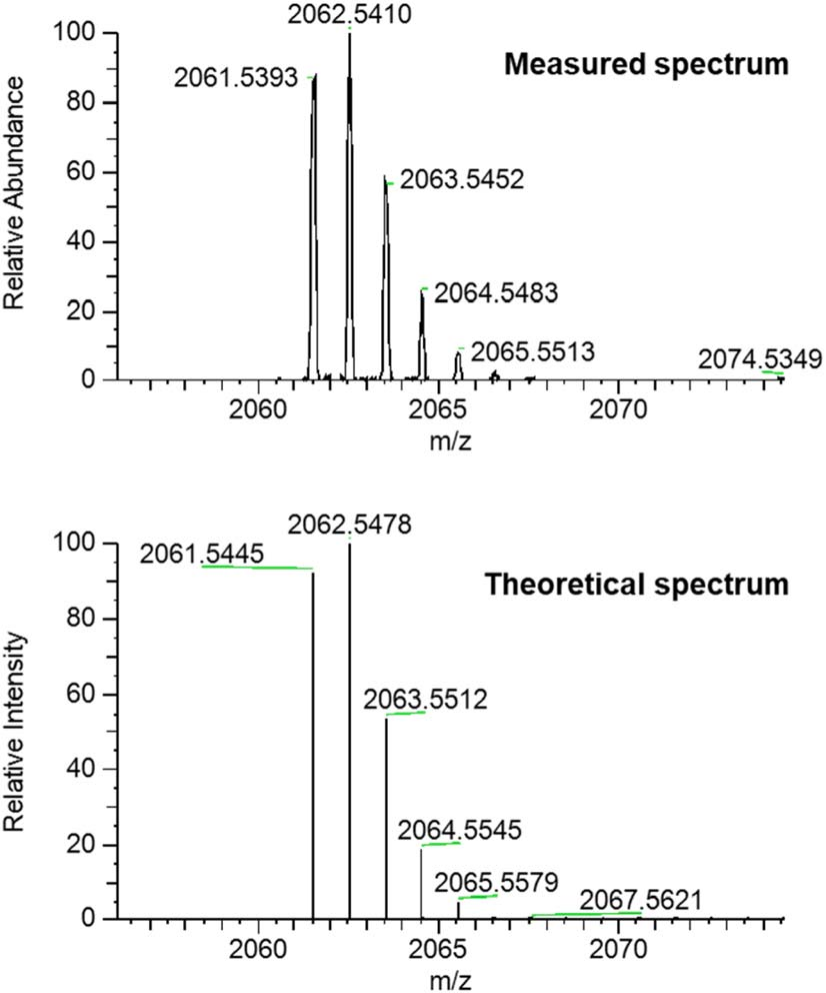
High resolution mass spectrum (ESI +ve) of [3]catenane 1 (top: measured; bottom: simulated).

**Fig. 3:**
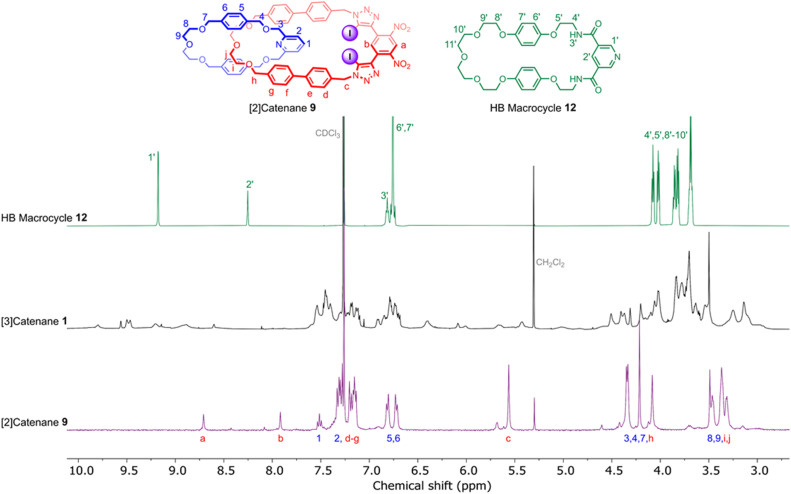
Stacked ^1^H NMR spectra of HB macrocycle 12 (top), [3]catenane 1 (middle) and [2]catenane 9 (bottom) (CDCl_3_, 500 MHz, 298 K).

The high resolution ESI +ve mass spectrum of 1 revealed the presence of a molecular ion peak with *m*/*z* = 2062.5374, which exhibited an isotope distribution in excellent agreement with the predicted chemical composition of the [3]catenane (Fig. 2). ^1^H NMR analysis of the compound in CDCl_3_ revealed a series of broad peaks, with the majority of proton signals falling in the 3.0–4.5 ppm and 6.5–7.75 ppm regions. A series of lower intensity signals were also observed further downfield (*δ* > 8.5 ppm), which are typical of dinitrobenzene and pyridyl proton signals (Fig. 3). The general distribution of the proton signals is therefore consistent with the functional groups present in the [3]catenane. However, the broad and coincident nature of the proton signals made definitive spectral assignment challenging (Fig. 3).

**Fig. 4:**
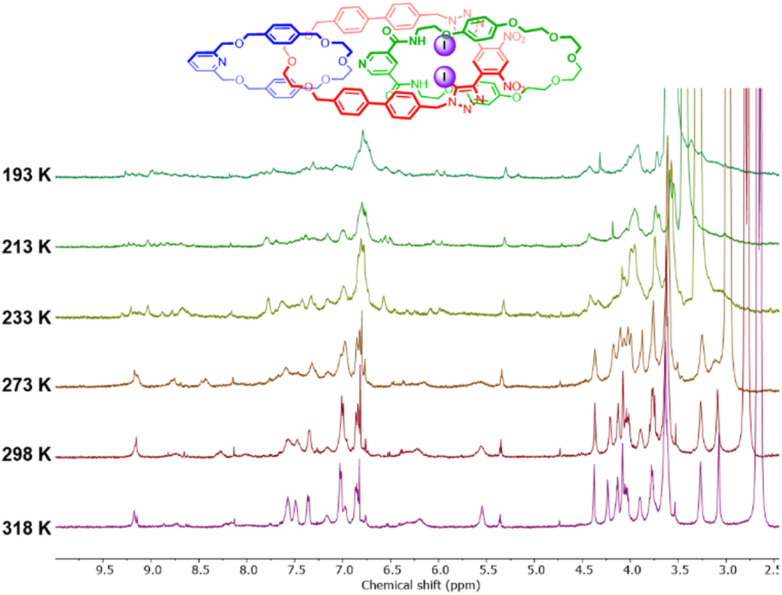
Variable temperature ^1^H NMR spectra of 1 (acetone-d_6_, 500 MHz).

Variable temperature NMR (VT-NMR) studies in CDCl_3_ and acetone-d_6_ were conducted in an attempt to improve the spectral resolution of the ^1^H NMR spectrum. Heating a solution of 1 in acetone-d_6_ to 318 K markedly reduced the linewidths of the proton signals (Fig. 4). In contrast, cooling the solution to 193 K resulted in further broadening and splitting of the proton resonances, which in some cases could not be distinguished from the baseline. This behaviour is wholly consistent with previous reports of dynamic higher-order [*n*]catenanes, which tend to exhibit split and broadened spectra at low temperatures due to the hindered co-conformational circum-rotation of the constituent macrocycles, whereas at higher temperatures, sharper proton signals are observed due to the rapid re-orientations of the macrocycles relative to the NMR timescale.^22^ In contrast, the ^1^H NMR spectrum of intermediate [2]catenane 9 did not display significant temperature dependence, suggesting the barrier to co-conformational circum-rotation is higher in 1, presumably due to the considerable steric clashes between the three interlocked ring components.

### ^1^H NMR cation and anion titration studies

Attention was then turned to investigating the guest binding properties of [3]catenane 1*via* a series of cation and anion titration studies. To this end, aliquots of NaBAr^F^_4_ were progressively added to a 1 mM solution of 1 in 1 : 1 CDCl_3_/CD_3_CN (v/v). With increasing concentration of the sodium cation guest, notable chemical shift perturbations were observed in the ^1^H NMR spectrum of 1, with a number of broad proton signals undergoing gradual splitting and sharpening (Fig. S10). Notably, the ethylene glycol protons in the 2.5–3.0 ppm region split and undergo an upfield shift with increasing Na^+^ concentration, which is diagnostic of alkali metal cation coordination by the oligo(ethylene glycol) units. The broad signals in the 9.0–9.5 ppm region resolve into a sharp peak, which has been assigned to the pyridyl protons of the 2,6-dimethanol pyridine unit in macrocycle 8. Taken together, these changes suggest that sodium cation binding to the [3]catenane gives rise to a co-conformationally “locked” alkali metal cation complex with sharp proton signals existing in fast exchange with the free receptor, with the former becoming the dominant species upon addition of excess sodium ions. However, the complexity of the spectral changes precluded a quantitative determination of the sodium cation association constant of 1. Anion titrations were conducted in an analogous fashion, with aliquots of TBACl progressively added to a 1 mM solution of [3]catenane 1 in 2.5% D_2_O/acetone-d_6_ and the resulting ^1^H NMR spectra recorded (Fig. S11). In this mixed aqueous/acetone solvent system, a number of well-resolved proton signals were visible, presumably due to the presence of water diminishing HB-driven co-conformational locking and therefore aiding in resolving the proton signals. With the addition of TBACl, some of these peaks broadened and vanished, while other signals that were previously indistinguishable from the baseline became well-resolved. Notably, the peaks that sharpened with increasing TBACl concentration could be assigned to protons proximal to the anion binding site (H_a_, H_b_ and H_c_ near the XB binding motif of central macrocycle 6). Notably, a signal which was assigned to H_2_ from the amide macrocycle sharpens and becomes visible upon addition of up to 1.0 equivalents of TBACl before broadening again. In contrast, protons H_b_ and either H_e_/H_f_ arising from the 2,6-dialkylpyridyl-containing macrocycle 8 broadened and vanished with increasing TBACl concentration. Analogous to observations made during Na^+^ binding experiments, the addition of a coordinating chloride anion is expected to “lock” the co-circum-rotation of the [3]catenane, leading to increased resolution of signals near the anion binding site whilst other regions of the [3]catenane remain co-conformationally labile, thereby giving rise to the observed linewidth changes in the ^1^H NMR spectrum.

### Single crystal X-ray diffraction studies

**Fig. 5:**
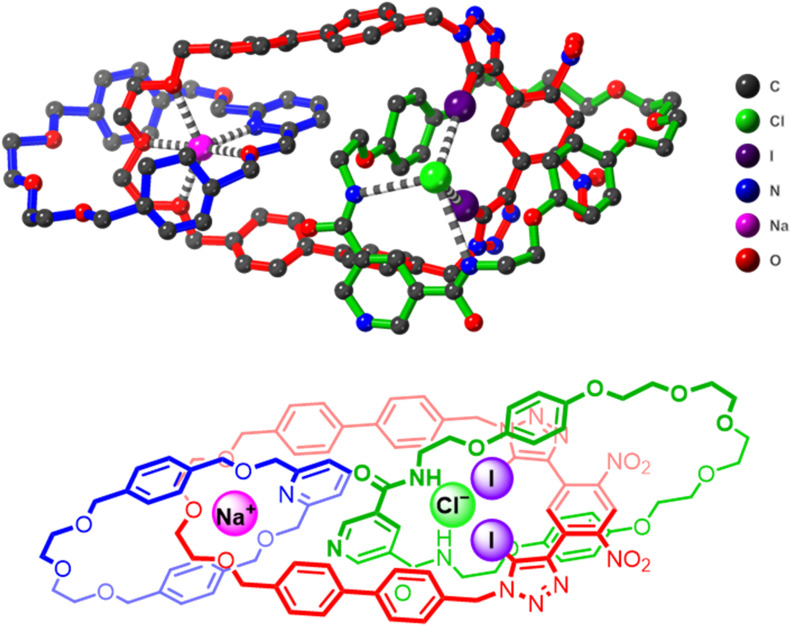
Solid-state XRD structure of [3]catenane 1 bound to a NaCl ion-pair in a host-separated binding mode. Hydrogen atoms have been omitted for clarity. Atom colours are as follows: black (carbon), blue (nitrogen), red (oxygen), purple (iodine), pink (sodium), green (chlorine). The chemical structure of the catenane is shown below for reference.

Unequivocal evidence for the successful formation and topology of the [3]catenane receptor 1 was established by single-crystal X-ray diffraction analysis. Small, needle-like orange crystals suitable for structural determination were obtained by slow evaporation of a titration solution of 1 containing 10 equivalents of NaBAr^F^_4_ in 1 : 1 CDCl_3_/CD_3_CN. The structure was solved and refined in the triclinic space group *P̄*1, revealing the heteroditopic [3]catenane receptor 1 encapsulating a spatially separated NaCl ion-pair (Fig. 5). The Cl^−^ anion presumably originates from trace chloride in the chlorinated solvent. The solid-state structure clearly validates the anticipated host-separated ion-pair binding mode of 1, with each ion precisely located within complementary interlocked cavities. Specifically, the Na^+^ cation is effectively coordinated through chelation involving the ethylene-glycol and pyridine motifs of the constituent macrocycles 6 and 8 respectively, (Na^+^/N(pyridine) 2.42, Na^+^/O(ether) 2.25–2.70 Å). On the other hand, the Cl^−^ anion is bound within the cavity of macrocycle 6, stabilized by distinct halogen bonding interactions with the iodo-triazole units (Cl^−^⋯I distances 3.58–3.16 Å) and hydrogen bonding contacts with the amide groups (Cl^−^⋯NH 3.39–3.61 Å). The observed robust binding of a Na^+^/Cl^−^ ion-pair by receptor 1 is remarkable given the high lattice enthalpy of NaCl, which strongly favours salt recombination rather than separated ion-pair encapsulation. Consequently, this represents one of the few examples of a heteroditopic receptor capable of binding a separated Na^+^/Cl^−^ ion-pair.^39–42^ To the best of our knowledge, Na^+^/Cl^−^ ion-pair binding by receptors using XB interactions as the primary anion binding interaction is unprecedented. The observed binding of Na^+^/Cl^−^ by [3]catenane 1 therefore provides promising evidence for the efficacy of using multiple higher order interlocked host cavities to enhance the ion-pair recognition properties of heteroditopic receptors.

## Conclusions

A heteroditopic [3]catenane ion-pair host system containing separate interlocked cavities for cation and anion binding has been synthesised in a stepwise manner utilising orthogonal cation and anion templates. The ^1^H-NMR signals of [3]catenane 1 showed temperature-dependent linewidths, in which the signals broaden and split at low temperatures while becoming sharper at higher temperatures, indicating hindered co-conformational circum-rotation of the three interlocked macrocycles. The addition of NaBAr^F^_4_ salt to the receptor led to a series of complex spectral changes in which several broad and overlapping proton signals split and become resolved, indicative of the alkali metal cation binding capability of the [3]catenane *via* its ethylene glycol units. The interlocked [3]catenane topology of 1 was confirmed by solid state single-crystal X-ray diffraction studies. Crucially, the crystal structure showed the [3]catenane binding to an NaCl ion-pair in a host-separated binding mode in which the two guest ions are encapsulated in their respective interlocked binding cavities, providing preliminary evidence for the potent ion-pair binding capabilities of the heteroditopic [3]catenane receptor. Ongoing work involves the investigation of alternative [3]catenane designs, in particular of the linker region in the central macrocycle, to improve the overall yield and NMR spectral resolution of the [3]catenane product. In summary, this study demonstrates the very first example of using orthogonal cation and anion templates in a stepwise and sequential strategy to direct the formation of higher-order heterocatenanes, notably comprising structurally distinct macrocycles. The application of this methodology is demonstrated with the preparation of a heteroditopic [3]catenane capable of NaCl ion-pair binding. Looking forward, the access to higher order heteroditopic interlocked architectures has a myriad of highly promising applications for selective ion-pair transport across biological membranes, organocatalysis mediated by ion-pairs and the selective sensing of ion-pairs, which is conceivably only possible with new synthetic routes for the preparation of higher-order interlocked molecules.

## Author contributions

H. M. T. performed the synthesis, and H. M. T. and A. D. the characterisation. H. M. T. performed the solid state single crystal X-ray diffraction data collection and A. J. M.-M. carried out the crystallographic analysis. All authors were involved in the drafting and revision of the manuscript. P. D. B. conceived and supervised the project.

## Conflicts of interest

There are no conflicts to declare.

## Supplementary Material

SC-017-D5SC05942A-s001

SC-017-D5SC05942A-s002

## Data Availability

Crystallographic data for 1·NaCl have been deposited at the CCDC under deposition number 2465955 and can be obtained *via*10.5517/ccdc.csd.cc2ns0yf.^43^ The data supporting this article have been included as part of the supplementary information (SI). Supplementary information is available. See DOI: https://doi.org/10.1039/d5sc05942a.
