# Supplementary material for: Orthogonal cation and anion template-directed synthesis of a heteroditopic [3]catenane for ion-pair recognition
Source: Chem Sci. 2026 Apr 2;17(21):10581–8. doi: 10.1039/d5sc05942a (PMC13088425; doi:10.1039/d5sc05942a)
Supplement: SC-017-D5SC05942A-s001 [file SC-017-D5SC05942A-s001.pdf]

Supplementary Information (SI) for Chemical Science.
This journal is © The Royal Society of Chemistry 2026

Supporting Information for

Orthogonal cation and anion template-directed synthesis of a heteroditopic [3]catenane for ion-pair recognition

Hui Min Tay, Andrew Docker, Antonio J. Martínez-Martínez, and Paul D. Beer

Contents

1 Materials and Methods 2
2 Synthesis and Characterization 3
3 ^{1}H NMR Guest Binding Studies 12
4 X-ray Crystallography........14
5 References........17

1 Materials and Methods

All solvents and reagents were purchased from commercial suppliers and used as received unless otherwise stated. Dry solvents were obtained by purging with nitrogen and then passing through an MBraun MPSP-800 column. H_2O was de-ionized and micro filtered using a Milli-Q® Millipore machine. Column chromatography was carried out on Merck® silica gel 60 under a positive pressure of nitrogen. Routine NMR spectra were recorded on either a Bruker AVIII 400, Bruker AVIII 500 or a Bruker AVIII 600 spectrometer with ^1H NMR titrations recorded on a Bruker AVIII 500 spectrometer. TBACl and $NaBAr^F$ were stored in a vacuum desiccator containing phosphorus pentoxide prior to use. Where mixtures of solvents were used, ratios are reported by volume. Chemical shifts are quoted in parts per million relative to the residual solvent peak. Mass spectra were recorded on a Bruker µTOF spectrometer.

Tris[(1-benzyl-1H-1,2,3-triazol-4-yl)methyl]amine (TBTA),[1] 6-dimethanolpyridine-functionalised macrocycles **4** and **8**,[2,3] 4,6-dinitro-1,3-bis(iodoethynyl)benzene **5**[4] and bis-amine **10**[5] were prepared according to previous literature reports. Macrocycle **12** was isolated as a by-product during the synthesis of **9** and its spectral analysis is in line with previously reported data.[5]

2 Synthesis and Characterization

Di(ethylene glycol)-based dichloride (2)

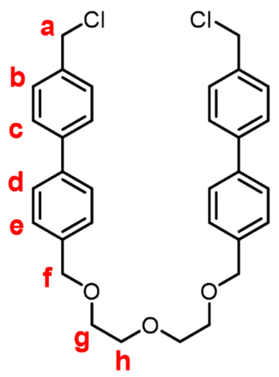

To a solution of diethylene glycol (1.0 mL, 11.1 mmol) in THF (100 mL), NaH (0.80 g, 33.3 mmol) was added portion-wise and the suspension was allowed to stir at room temperature for 20 minutes. 4,4'-bis(chloromethyl)-biphenyl (11.2 g, 44.5 mmol) was added and the reaction mixture was stirred at reflux for 24 hours, after which the precipitate was removed by filtration, and the filtrate concentrated *in vacuo.* The residue was redissolved in CH_2Cl_2 (100 mL), the organic layer washed with H_2O (2 × 100 mL) and brine (2 × 100 mL), dried over $MgSO_4$ and the solvent removed under vacuum. Purification by silica gel column chromatography (eluent: 2% EtOAC/CH_2Cl_2) afforded the dichloride **2** as a white solid (1.90 g, 32%).

^{1}H NMR (500 MHz, $CDCl_3$) δ 7.55 (m, 8H, $H_{c,d}$), 7.43 (m, 8H, $H_{b,e}$), 4.63 (s, 4H, $H_{a/f}$), 4.63 (s, 4H, $H_{a/f}$), 3.74 – 3.69 (m, 8H, $H_{g,h}$).

^{13}C NMR (126 MHz, $CDCl_3$) δ 141.23, 139.93, 137.85, 136.59, 129.21, 128.38, 127.55, 127.25, 73.09, 70.93, 69.75, 46.19.

HRMS (ESI +ve) m/z: 535.1809 ($[M+H]^+$, $C_{32}H_{33}Cl_2O_3$ requires 535.1801).

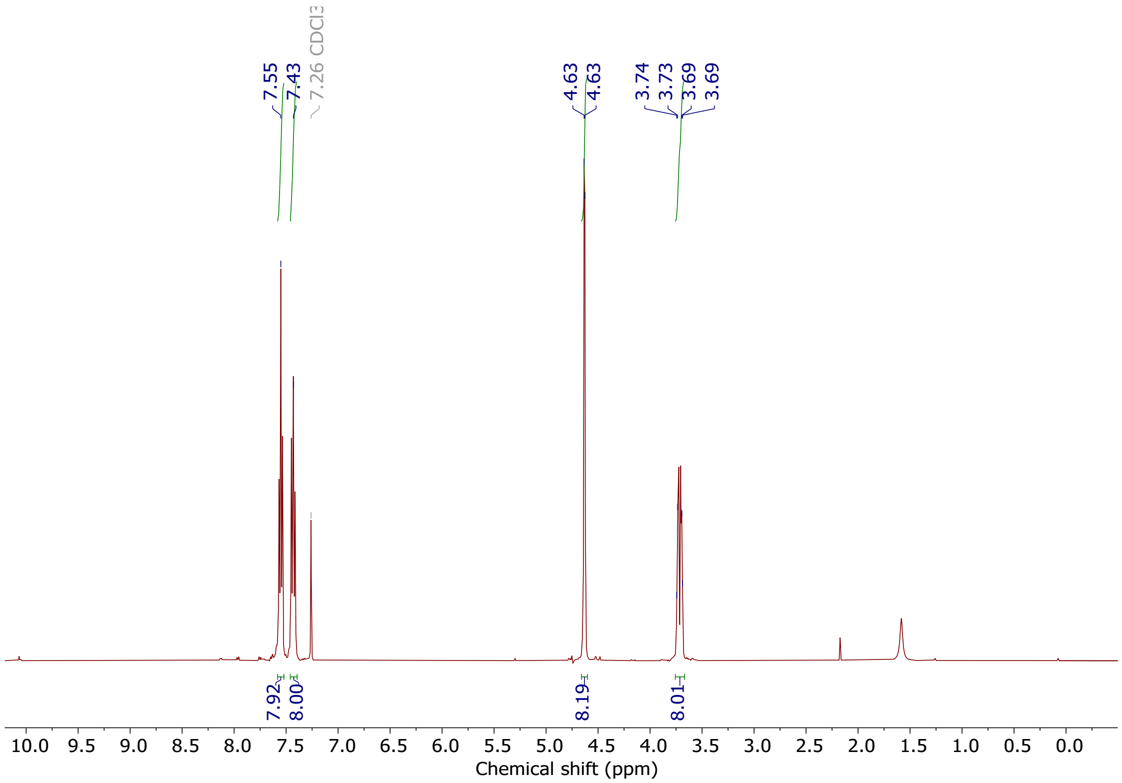

*Figure S 1. ^{1}H NMR spectrum of di(ethylene glycol)-based dichloride **2** ($CDCl_3$, 500 MHz, 298 K).*

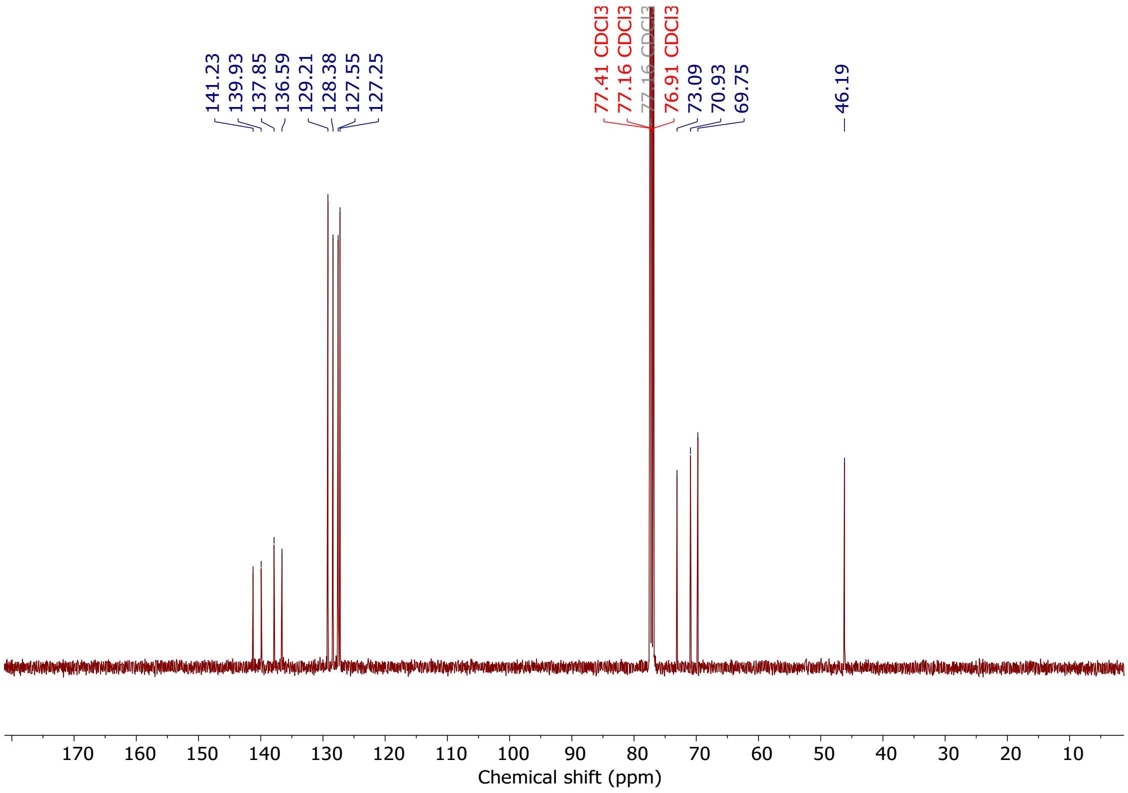

*Figure S 2. ^{13}C NMR spectrum of di(ethylene glycol)-based dichloride **2** ($CDCl_3$, 126 MHz, 298 K).*

Di(ethylene glycol)-based bis-azide (3)

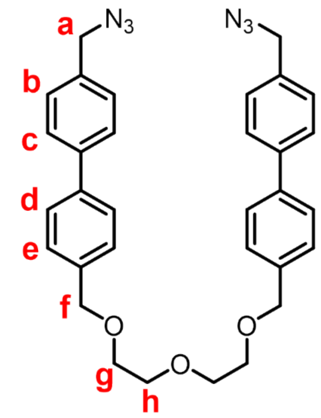

Dichloride **2** (1.0 g, 1.87 mmol) was dissolved in DMSO (9 mL). Sodium azide (0.49 g, 7.48 mmol) was added and the reaction mixture was stirred at room temperature overnight. The crude reaction mixture was diluted with H_2O (50 mL) and extracted with diethyl ether (30 mL × 3). The combined organic layer was washed with brine (30 mL × 3), dried over anhydrous $MgSO_4$, filtered and concentrated in vacuo to afford **3** as a white solid (0.95 g, 93%).

^{1}H NMR (500 MHz, $CDCl_3$) δ 7.64 (d, J = 8.4 Hz, 4H, H_d), 7.60 (d, J = 8.2 Hz, 4H, H_c), 7.48 (d, J = 8.2 Hz, 4H, H_e), 7.42 (d, J = 8.2 Hz, 4H, H_b), 4.68 (s, 4H, H_f), 4.43 (s, $4H_a$), 3.79-3.74 (m, 8H, $H_{g,h}$).

^{13}C NMR (126 MHz, $CDCl_3$) δ 141.08, 139.93, 137.80, 134.48, 128.78, 128.37, 127.60, 127.21, 73.07, 70.90, 69.72, 54.66.

HRMS (ESI +ve) m/z: 549.2615 ($[M+H]^+$, $C_{32}H_{33}N_6O_3$ requires 549.2609).

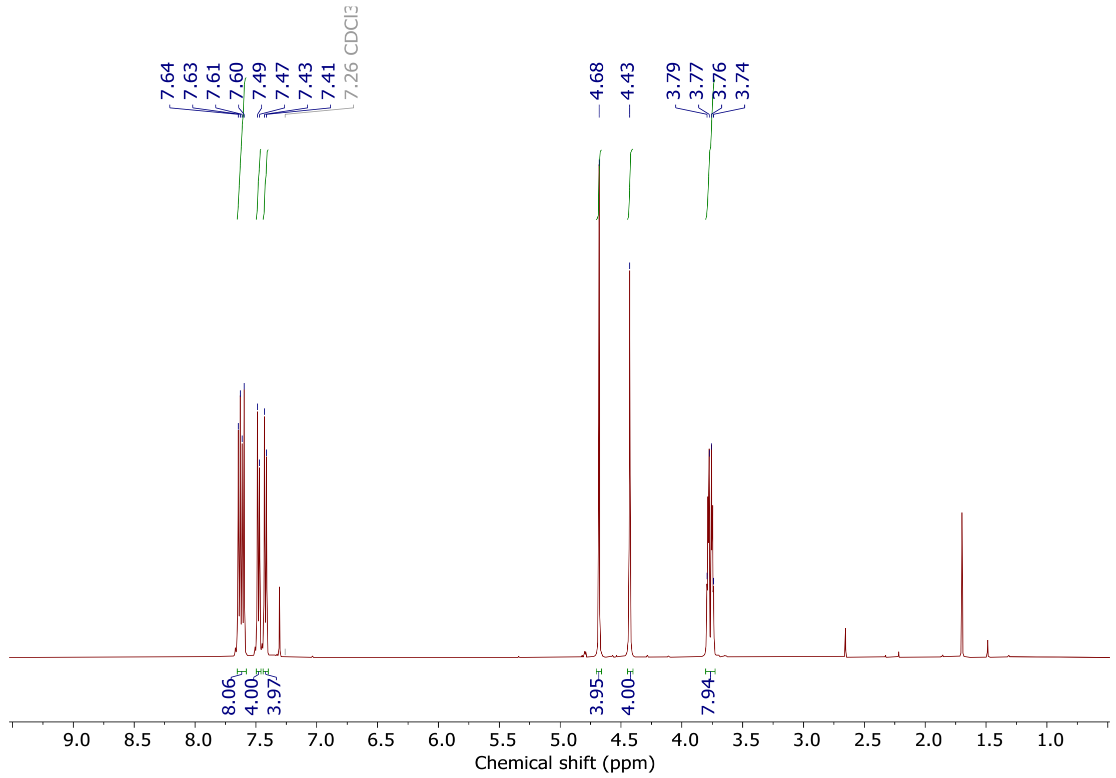

*Figure S 3. ^{1}H NMR spectrum of di(ethylene glycol)-based bis-azide **3** ($CDCl_3$, 500 MHz, 298 K).*

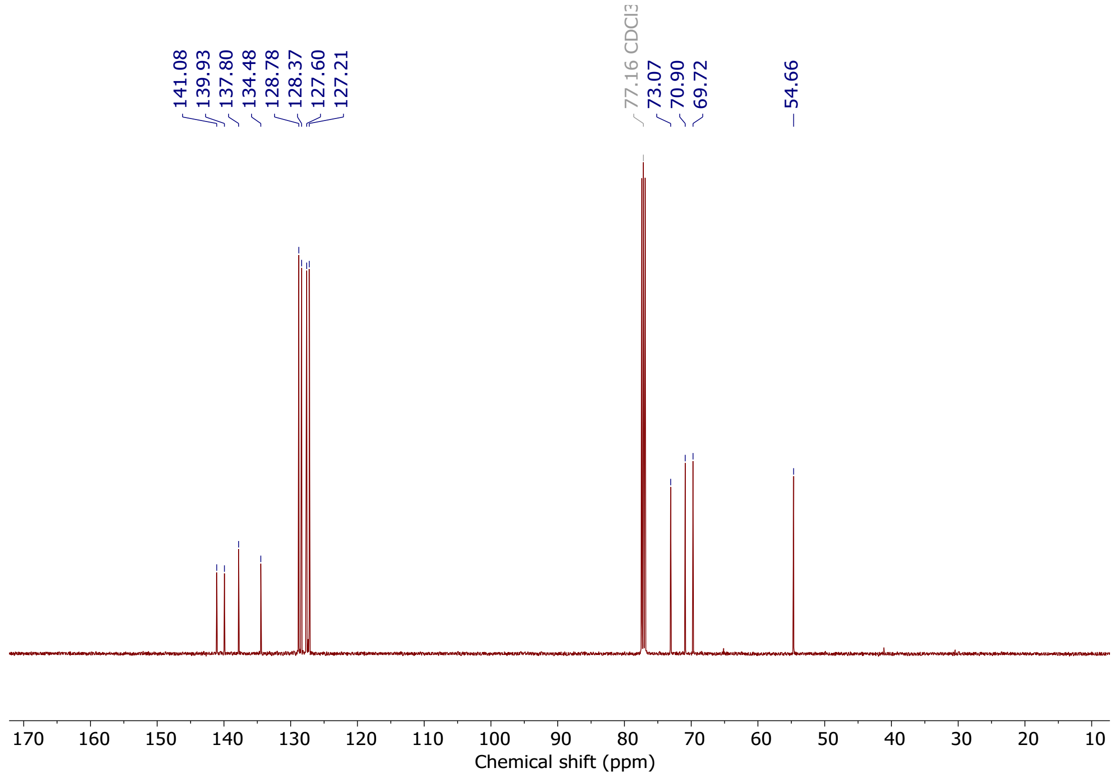

*Figure S 4. ^{13}C NMR spectrum of di(ethylene glycol)-based bis-azide **3** ($CDCl_3$, 126 MHz, 298 K).*

Hetero[2]catenane (9)

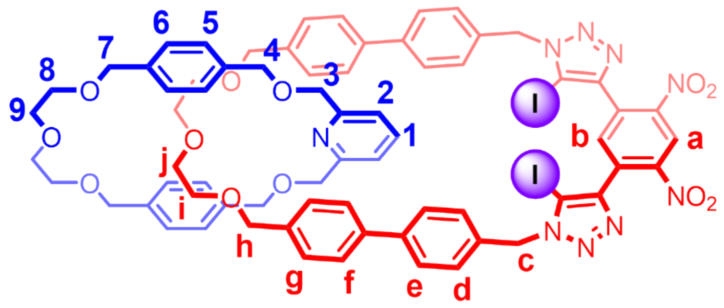

Macrocycle **8** (40.0 mg, 0.088 mmol) and NaBAr^F (78.8 mg, 0.088 mmol) were dissolved in dry, degassed CH_2Cl_2 (4 mL) and stirred for 30 minutes at room temperature. A solution of bis-azide **3** (47.6 mg, 0.088 mmol) in CH_2Cl_2 (2 mL) was added and the mixture stirred for a further 30 minutes. A solution of bis(iodoalkyne) **5** (41.6 mg, 0.088 mmol) in CH_2Cl_2 (2 mL) was added, followed by a dropwise addition of a premixed solution of $[Cu(CH_3CN)_4]PF_6$ (16.4 mg, 0.044 mmol) and TBTA (23.6 mg, 0.044 mmol) in CH_2Cl_2 (4 mL). The reaction mixture was stirred at room temperature in the dark for 48 hours, then was diluted with CH_2Cl_2 (40 mL). The organic layer was washed with EDTA/NH_4OH (2 × 25 mL) and H_2O (2 × 25 mL), dried over $MgSO_4$, filtered and concentrated under vacuum. The crude was purified by iterative preparative TLC purification (eluent: 60:40:1 CH_2Cl_2/EtOAc/MeOH, then 2.5% MeOH/CH_2Cl_2), afforded [2]catenane **9** as an orange solid (18 mg, 14%).

^{1}H NMR (500 MHz, acetone-d_6) δ 8.81 (s, 1H, H_a), 8.07 (s, 1H, H_b), 7.67 (t, J = 7.8 Hz, 1H, H_1), 7.43 (m, 8H, $H_{e,f}$), 7.25 (m, 10H, $H_{d,g,2}$), 6.90 (d, J = 8.0 Hz, 4H, H_5), 6.81 (d, J = 8.0 Hz, 4H, H_6), 5.78 (s, 4H, H_c), 4.39 (s, 4H, H_4), 4.33 (s, 4H, H_h), 4.27 (s, 4H, H_3), 4.10 (s, 4H, H_7), 3.35-3.27 (m, 16H, $H_{i,j,8,9}$).

^{13}C NMR (151 MHz, acetone-d_6) δ 158.93, 149.63, 147.14, 141.48, 139.95, 139.16, 138.41, 137.98, 137.61, 136.96, 134.37, 130.19, 129.36, 129.10, 129.01, 128.43, 127.80, 123.00, 120.38, 82.72, 73.61, 73.11, 72.99, 72.90, 72.44, 71.15, 71.06, 70.13, 69.77, 54.88.

HRMS (ESI +ve) m/z: 1466.2897 ($[M+H]^+$, $C_{69}H_{66}N_9O_{12}I_2$ requires 1466.2915).

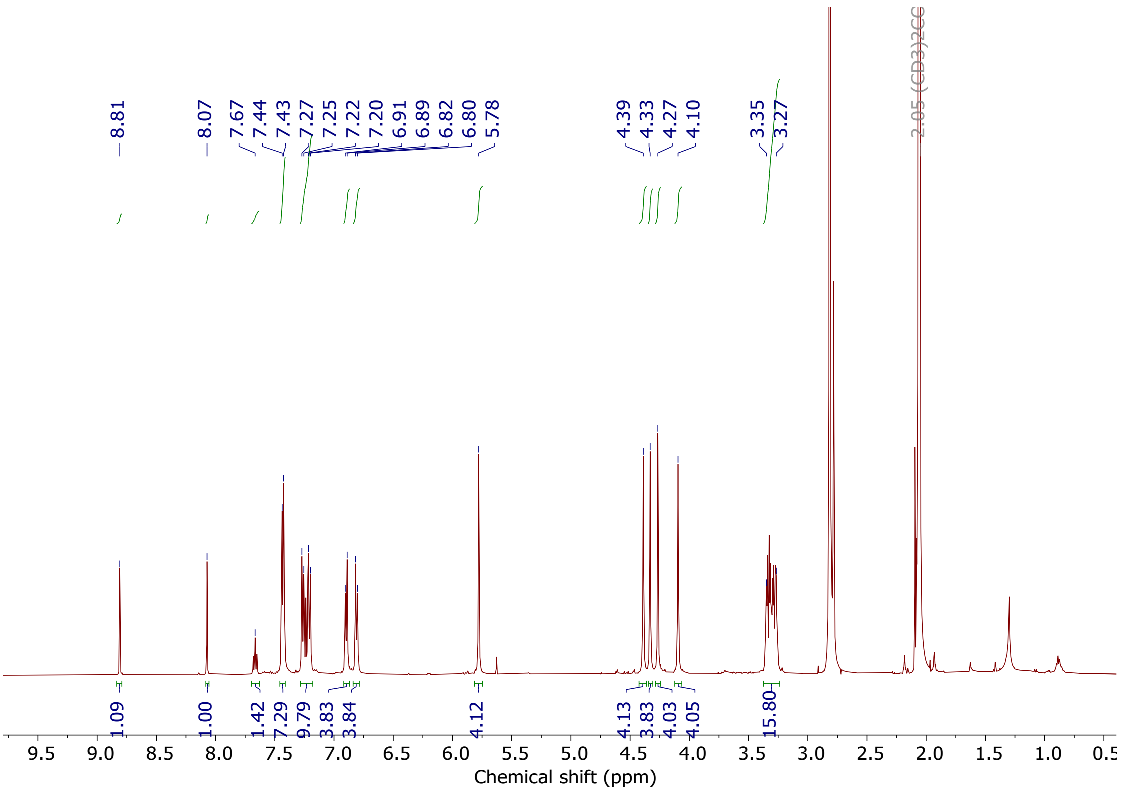

*Figure S 5. ¹H NMR spectrum of [2]catenane **9** (acetone-d$_6$, 500 MHz, 298 K).*

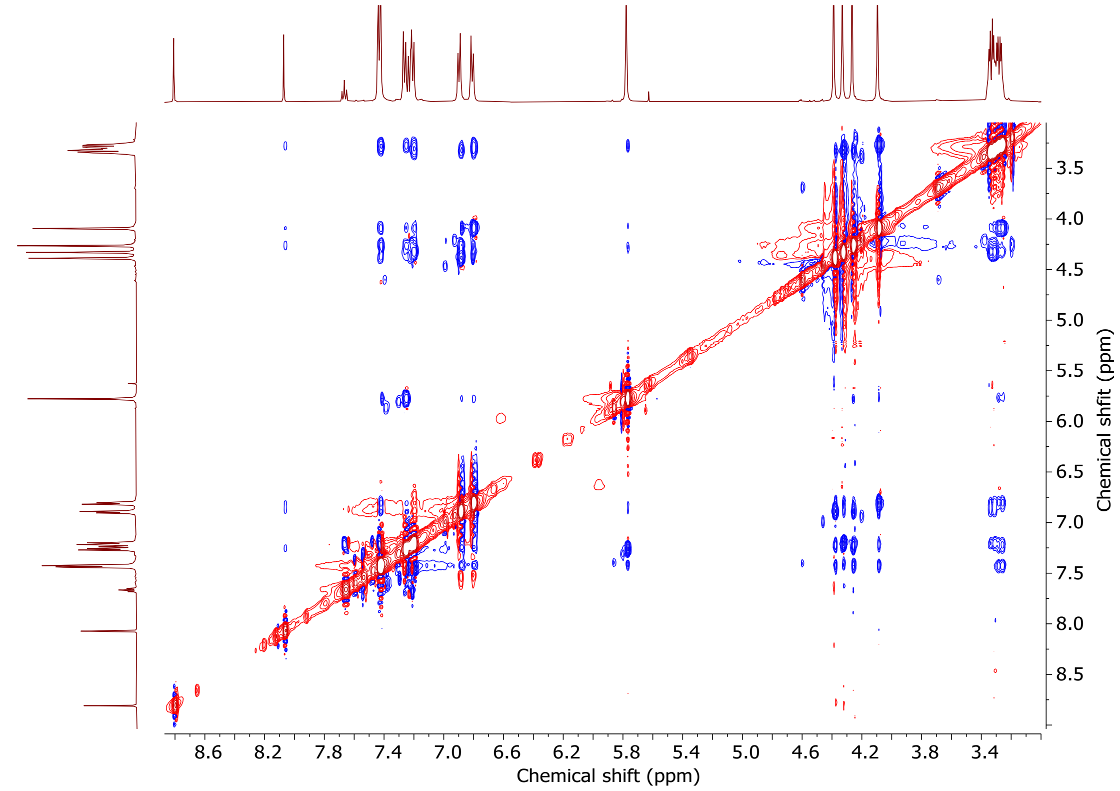

*Figure S 6. ¹H-¹H 2D ROESY NMR spectrum of [2]catenane **9** (acetone-d$_6$, 500 MHz, 298 K).*

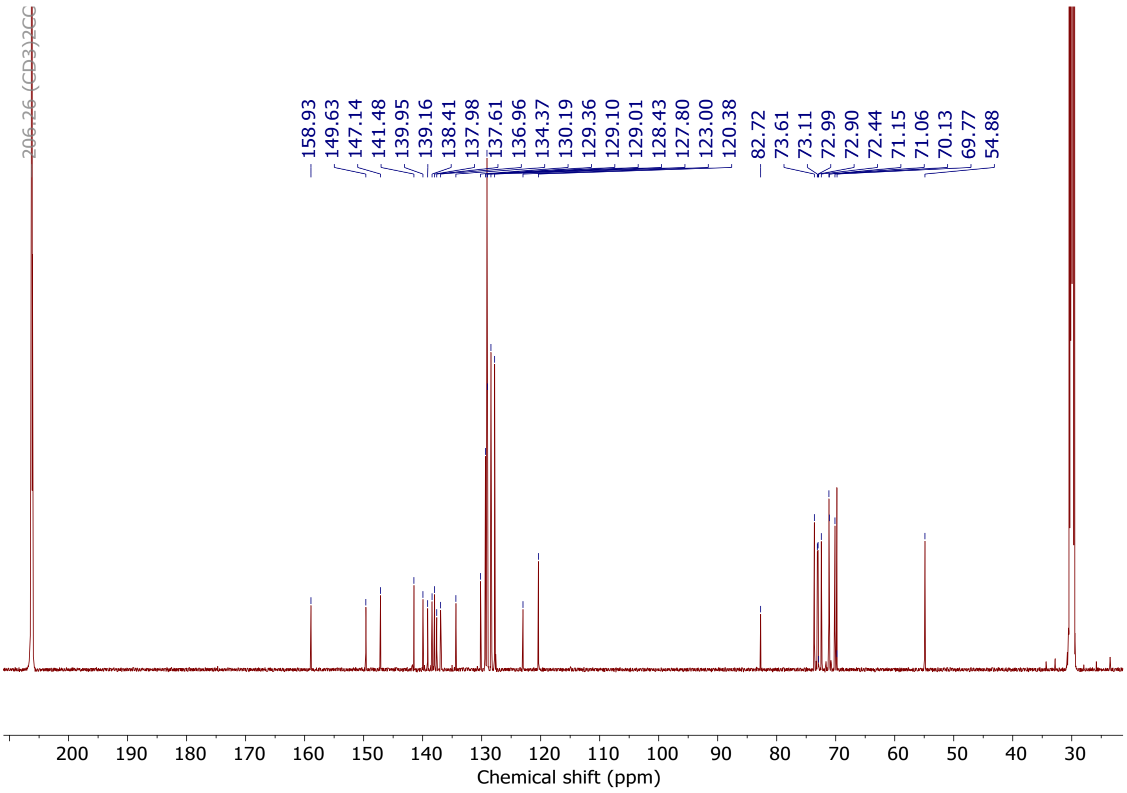

*Figure S 7. ^{13}C NMR spectrum of [2]catenane **9** (acetone-d$_6$, 126 MHz, 298 K).*

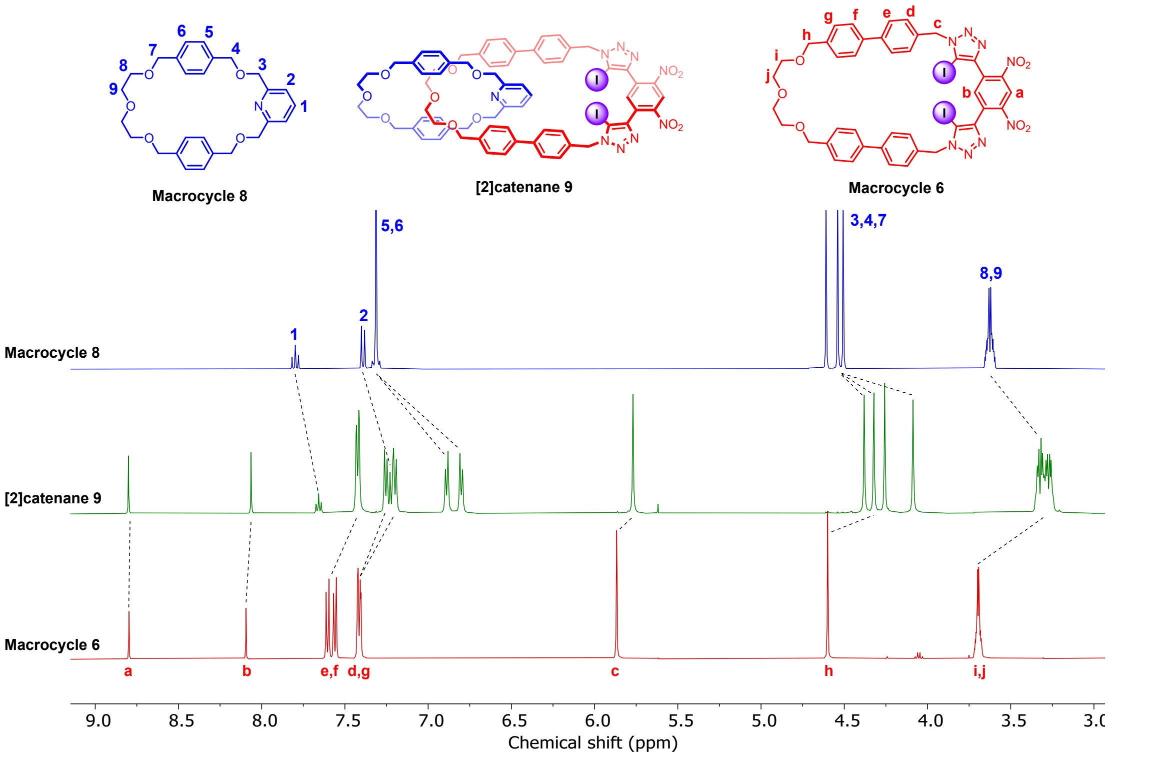

*Figure S 8. Stacked ^{1}H NMR spectra of macrocycle **8** (top), [2]catenane **9** (middle) and XB macrocycle **6** (bottom) (500 MHz, acetone-d$_6$, 298 K).*

Expanded Spectrum RT 0.18, NL 49700568, Peak [1], Target Mass 1466.2915

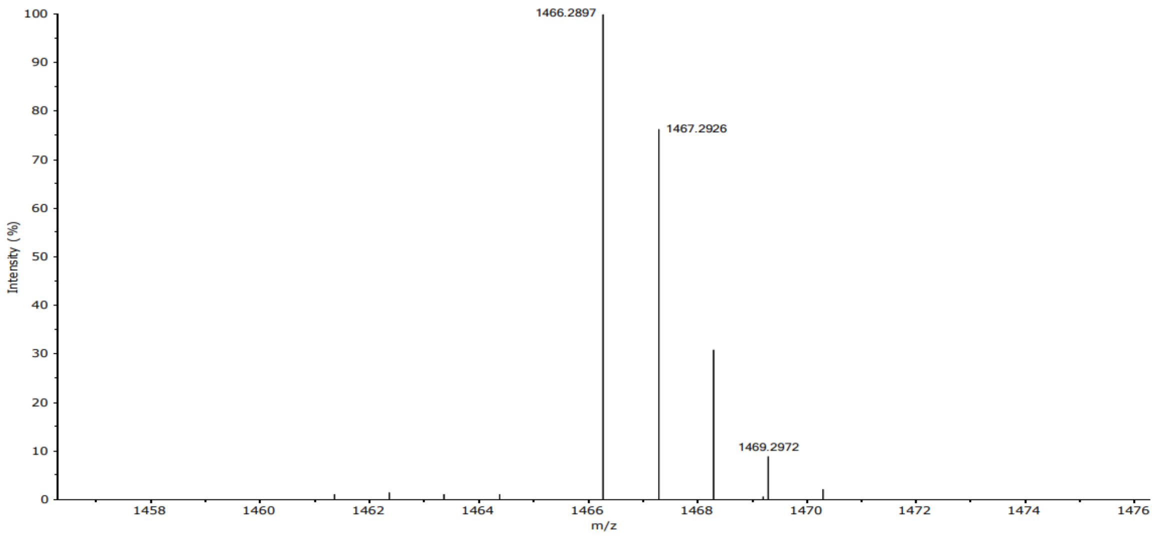

Theoretical Spectrum for C69H66I2N9O12, Minimum Abundance 0.01%

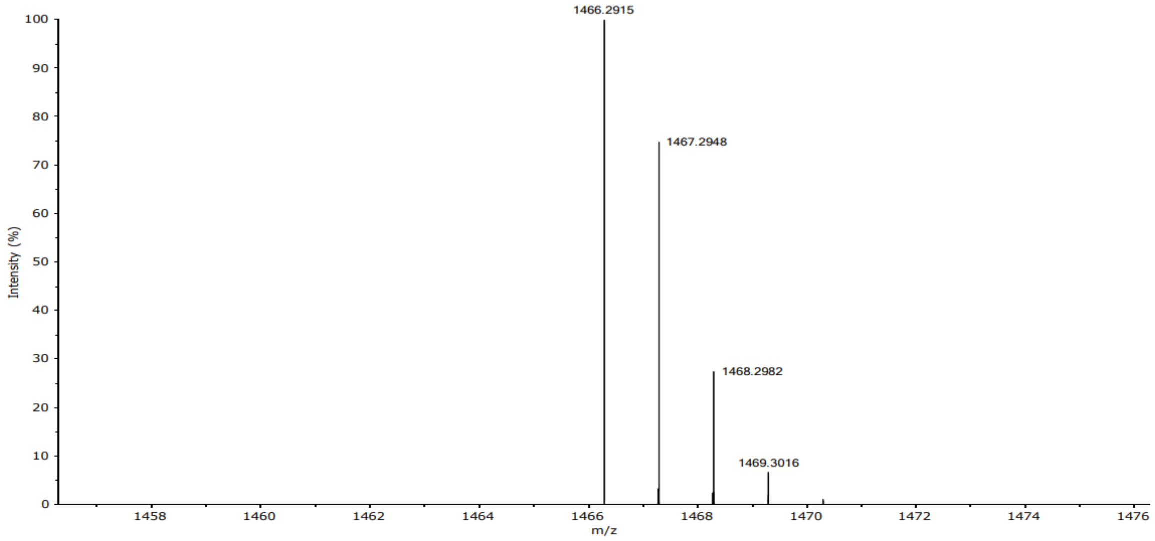

*Figure S 9. High-resolution ESI+ve mass spectrum of [2]catenane **9** (Top: measured spectrum. Bottom: theoretical spectrum).*

Hetero[3]catenane (1)

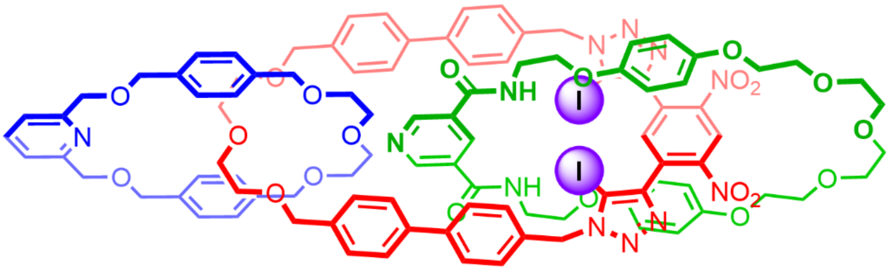

3,5-Pyridinedicarboxylic acid (17 mg, 0.10 mmol) was suspended in neat oxalyl chloride (1 ml), to which was added one drop of DMF. After stirring for 2 hours, the mixture became homogenous, at which point the volatiles were removed in vacuo to afford the corresponding bis-acid chloride **11**. In a separate flask, [2]catenane **9** (15 mg, 0.010 mmol), bis-amine **10** (48 mg, 0.10 mmol), TBACl (2.9 mg, 0.010 mmol) and NEt_3 (0.05 mL) were dissolved in anhydrous CH_2Cl_2 (5 mL) and cooled to 0 °C. Bis-acid chloride **11** was dissolved in anhydrous CH_2Cl_2 (1.5 mL) and added dropwise to reaction mixture, after which the mixture was allowed to warm to room temperature and stirred overnight. The reaction mixture was diluted with CH_2Cl_2 (50 mL) and washed with sat. K_2CO_3 (aq) (2 × 30 mL) and H_2O (2 × 30 mL), the collected organic phase was dried over $MgSO_4$, concentrated to dryness *in vacuo* and purified by preparative thin layer chromatography (50:50:4 CH_2Cl_2/EtOAc/MeOH) to yield [3]catenane **1** as a yellow solid (2.5 mg, 12%).

^{1}H NMR and **^{13}C NMR** spectral data could not be obtained due to the low yield of **4.10** as well as broadening of NMR resonances (see manuscript for details).

HRMS (ESI +ve) m/z: 2062.5374 ($[M+H]^+$, $C_{100}H_{103}N_{12}O_{21}I_2$ requires 2062.5478).

3 ^{1}H NMR Guest Binding Studies

^{1}H NMR titration experiments were performed on a Bruker Avance III NMR 500 MHz spectrometer (AVD500) at 298 K. In a typical experiment, aliquots of a 50 mM stock solution containing the guest ion (TBACl or NaBAr^F) were added to 0.5 mL of a 1.0 mM solution of [3]catenane **1**, where 1.0 equivalent of guest ion added corresponds to 10.0 µL of the stock solution. The samples were thoroughly mixed before recording the ^{1}H NMR spectra. 17 spectra corresponding to 0.0, 0.2, 0.4, 0.6, 0.8, 1.0, 1.2, 1.4, 1.6, 1.8, 2.0, 2.5, 3.0, 4.0, 5.0, 7.0 and 10.0 equivalents of added guest were obtained.

The broad signals in the NaBAr^F titration and the appearance of a second set of peaks in the TBACl titration (presumably arising from slow exchange in the mixed aqueous/acetone solvent system) precluded a quantitative determination of association constants. Nonetheless, the observed changes in chemical shift/linewidth of the various proton resonances are indicative of a binding interaction between the [3]catenane and the cation/anion guests.

Titration of [3]catenane 1 with NaBAr^F

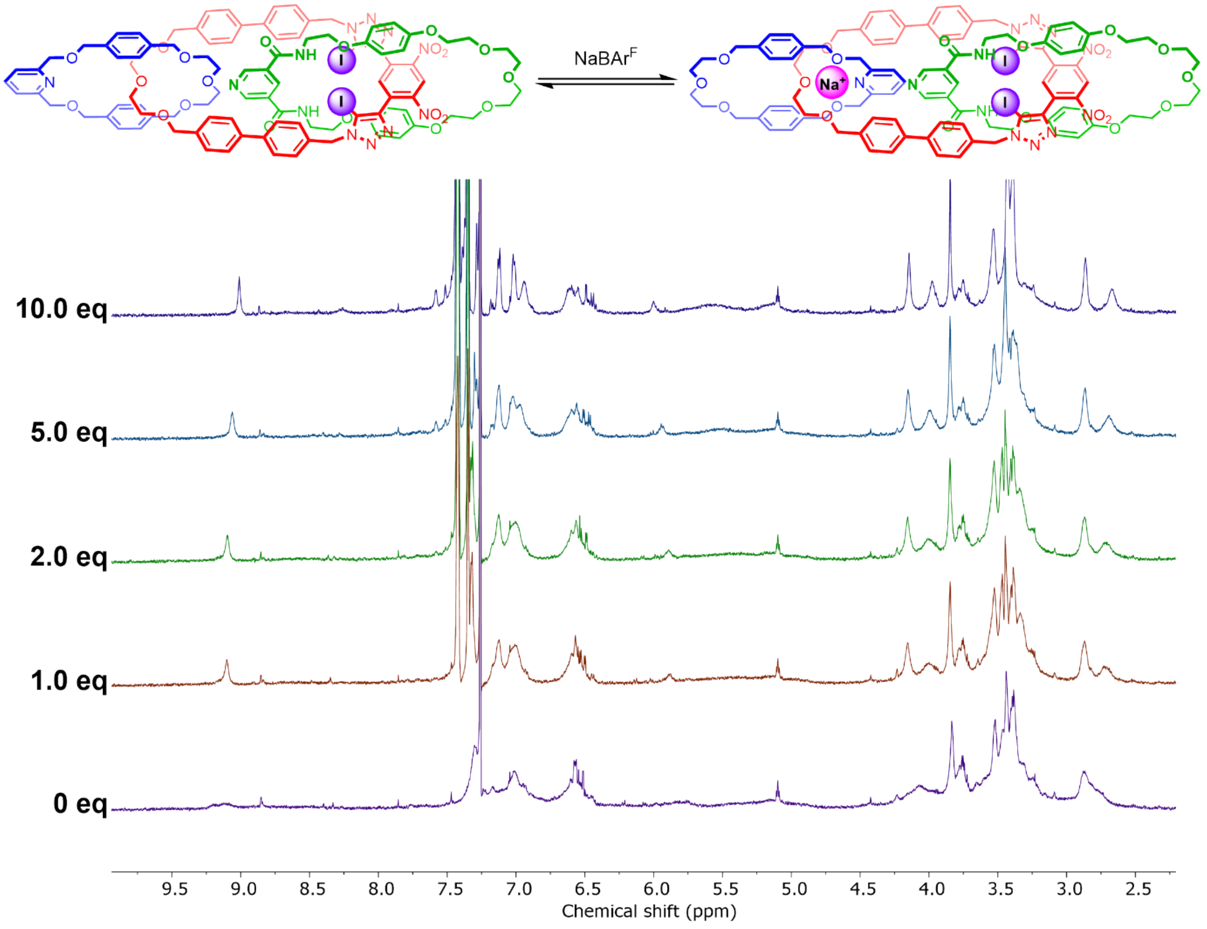

*Figure S 10. Overlaid ^{1}H NMR titration spectra of **1** upon progressive addition of 10 eq. NaBAr^F (500 MHz, 1:1 $CD_3CN/CDCl_3$, 298 K).*

Titration of [3]catenane 1 with TBACl

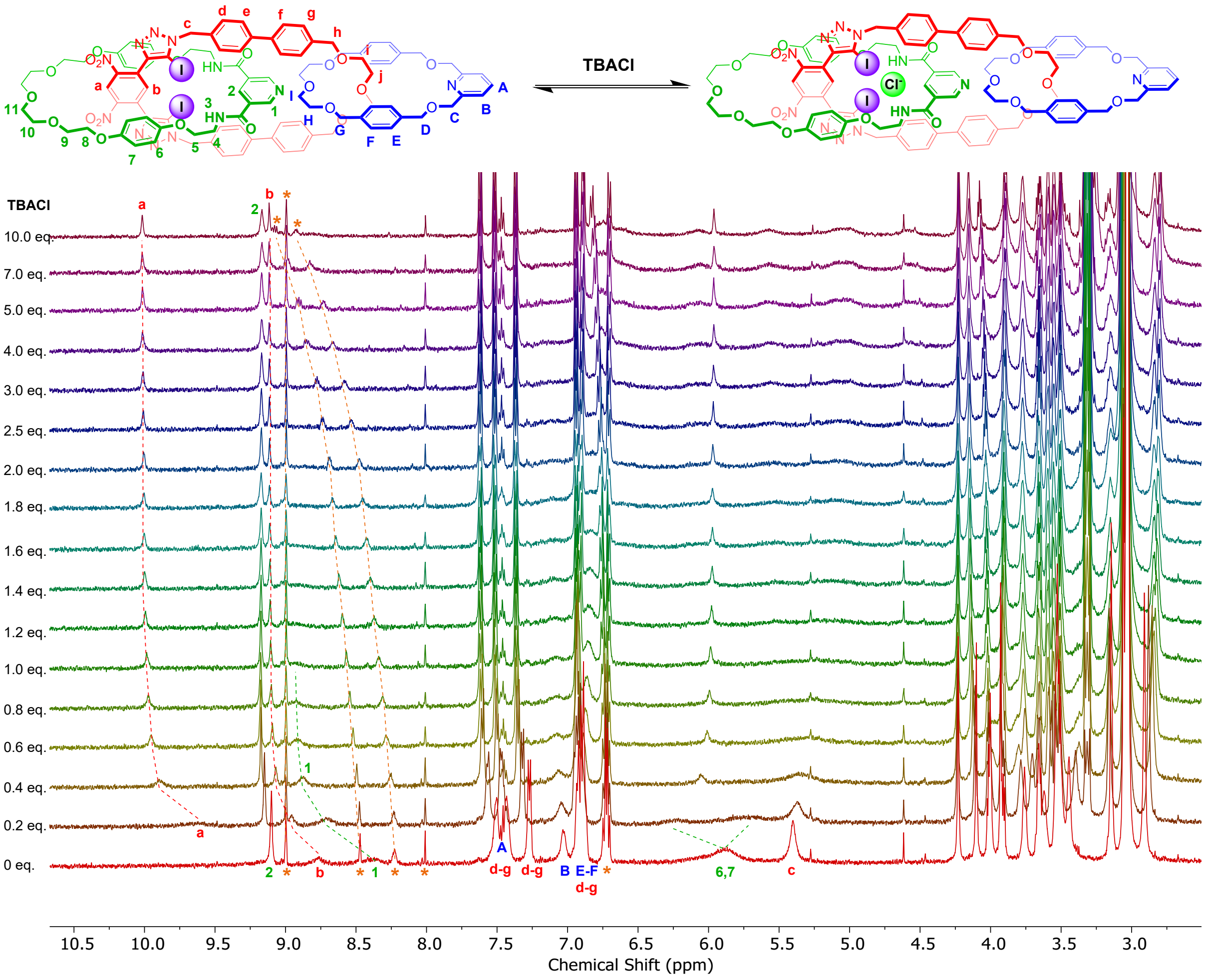

*Figure S 11. Overlaid ^{1}H NMR titration spectra of **1** upon progressive addition of 10 eq. TBACl (500 MHz, 2.5% D_2O/acetone-d_6, 298 K).*

4 X-ray Crystallography

Crystal structure determinations

Single-crystal X-ray diffraction data was collected under a stream of nitrogen gas using an Oxford Cryostream unit[6] at the given temperature with a four-circle Diffraction data was reduced and processed using CrysAlisPro package.[7] The structures were solved using SHELXT[8] and refined to convergence on F^2 and against all independent reflections by full-matrix least-squares using SHELXL[9] in combination with the GUI OLEX2[10] program. All non-hydrogen atoms were refined anisotropically, and hydrogen atoms were geometrically placed unless otherwise stated (see specific details in the text) and allowed to ride on their parent atoms. Distances and angles were calculated using the full covariance matrix. Selected crystallographic data are summarized in the text and full details are given in the supplementary deposited CIF files (CCDC 2465955). These data can be obtained free of charge from the Cambridge Crystallographic Data Centre via http://optimized.ccdc.cam.ac.uk/data_request/cif.

Selected crystallographic and refinement data

Table S1. Crystal data for **1** (CCDC 2465955)

Empirical formula	$C_{100}H_{10}ClI_2N_{12}NaO_{21}$	
Formula weight	2120.17	
Temperature	150 K	
Wavelength	1.54184 Å	
Crystal system	Triclinic	
Space group	P-1	
Unit cell dimensions	a = 15.4730(4) Å	α = 83.851(2)°.
	b = 13.7427(2) Å	β = 85.045(2)°.
	c = 15.8524(5) Å	γ = 73.865(3)°.
Volume	10430.8(5) Å^3	
Z	4	
Density (calculated)	1.350 Mg/m^3	
Absorption coefficient	5.610 mm^{-1}	
F(000)	4352	
Crystal size	0.15 x 0.08 x 0.07 mm^3	
Theta range for data collection	3.673 to 40.792°.	
Index ranges	-13<=h<=13, -13<=k<=13, -37<=l<=37	
Reflections collected	114058	
Independent reflections	13159 [R(int) = 0.1444]	
Completeness to theta = 40.792°	99.1%	
Absorption correction	Semi-empirical from equivalents	
Refinement method	Full-matrix least-squares on F^2	
Data / restraints / parameters	13159 / 4881 / 2467	

Goodness-of-fit on F^2	1.782
Final R indices [I>2sigma(I)]	R1 = 0.1464, wR2 = 0.4201
R indices (all data)	R1 = 0.1722, wR2 = 0.4436
Extinction coefficient	n/a
Largest diff. peak and hole	1.550 and -0.875 e.$Å^{-3}$

Additional details for 1: Due to inherent limitations associated with very small crystal size, weak diffraction intensity, and significant positional disorder with modulation effects, structural refinement of this large [3]catenane **1** was intrinsically challenging, limiting the data resolution to 1.18 Å (maximum sin(θ)/λ = 0.4237). Consequently, a lower-than-ideal data-to-parameter ratio (5.33), elevated R-values (R1 = 14.6%; wR2 = 44%), and reduced precision in bond distances were obtained. Specific alerts and several statistical reflection outliers were thoroughly investigated and found to originate from these inherent structural and experimental constraints rather than systematic errors or incorrect symmetry assignments. Additionally, low-angle reflections were partially obscured by the beamstop due to the large unit cell dimensions. Despite these limitations, comprehensive refinement and careful validation ensured the reported structural model represents the best achievable solution, reliably capturing the essential chemical and structural characteristics of this complex supramolecular assembly. This crystal structure determination undoubtedly serves as unequivocal proof of the chemical identity and connectivity of the targeted [3]catenane molecule **1**.

Alert level A

THETM01_ALERT_3_A The value of sine(theta_max)/wavelength is less than 0.550Calculated sin(theta_max)/wavelength = 0.4237

Author Response: The crystal diffracted to 1.18 Ang. This is a typical value for such large molecules incorporating disordered solvent. Due to intrinsic sample limitations, including very small crystal size, weak diffraction intensity, and significant solvent disorder, data collection beyond a resolution limit of 1.18 Ang. was not achievable. Consequently, the maximum sin(theta)/lambda value obtained was 0.4237. Extensive experimental efforts confirmed this as the practical diffraction limit for this structurally complex [3]catenane.

PLAT088_ALERT_3_A Poor Data / Parameter Ratio .... 5.33 Note

Author Response: This is a typical value for such large molecules incorporating disordered solvent. The presence of two large independent [3]catenane molecules within the asymmetric unit (space group P-1) inherently required the refinement of a large number of parameters. Moreover, the crystal exhibited modulated diffraction patterns, and as the model do not reflect such modulation, this result with unresolved long-range disorder observing two molecules within the asymmetric unit that significantly complicated the refinement. This required the application of numerous restraints and constraints, thereby lowering the data-to-parameter ratio to 5.33. Further reduction in refined parameters (e.g., using riding hydrogen models) was explored but found practically unfeasible given the structural complexity and intrinsic disorder.

Alert level B

PLAT084_ALERT_3_B High wR2 Value (i.e. > 0.25) .... 0.44 Report

Author Response: This is a typical value for such large molecules incorporating disordered solvent. The elevated wR2 value (0.44) arises primarily from inherent structural complexity and modulation, extensive positional disorder, and significant residual electron density due to weak diffraction (resolution limit of 1.18 Angs.). Exploration of alternative space groups, twin models, and refinement strategies did not yield improved results. Despite extensive use of restraints and constraints, these intrinsic structural and data limitations could not be fully resolved, thus accounting for the increased wR2 value.

PLAT342_ALERT_3_B Low Bond Precision on C-C Bonds .... 0.03761 Ang.

Author Response: Reduced bond precision is directly attributable to limited resolution (1.18 Ang), weak diffraction intensity, significant positional disorder, and modulation effects inherent in this complex [3]catenane structure.

PLAT369_ALERT_2_B Long C(sp2)-C(sp2) Bond C60 - C63 . 1.57 Ang.

Author Response: This elongated C-C bond distance arises from significant positional disorder and modulation within the structure, exacerbated by weak diffraction intensity and limited data resolution (1.18 Ang). Despite careful refinement, this bond could not be modeled more precisely under these experimental constraints.

PLAT411_ALERT_2_B Short Inter H...H Contact H11L ..H14A . 1.95 Ang. x,-1+y,1+z = 1_546 Check

Author Response: This short intermolecular H...H contact results from unresolved positional disorder and modulation inherent to this structurally complex [3]catenane, coupled with limited diffraction quality and resolution (1.18 Ang). Extensive refinement with appropriate restraints and constraints confirms that symmetry assignment and molecular placement are correct and represent the best achievable model under these constraints.

PLAT910_ALERT_3_B Missing # of FCF Reflection(s) Below Theta(Min). 13 Note 1 0 0, 0 1 0, 1 1 0, 0 -1 1, -1 0 1, 0 0 1, 1 0 1, 0 1 1, 1 1 1, 0 0 2, 1 0 2, 0 1 2, 0 0 3,

Author Response: Missing low-angle reflections are due to beam stop shadowing caused by the large unit cell dimensions and experimental set-up constraints. This did not impact the overall structural refinement or interpretation.

PLAT934_ALERT_3_B Number of (Iobs-Icalc)/Sigma(W) > 10 Outliers. 8 Check -2 5 0, -1 -4 2, 2 -2 6, -2 0 7, -4 5 9, 0 1 12, 0 3 16, 3 -2 21,

Author Response: These statistical outliers result from weak diffraction intensity, significant structural modulation, and positional disorder intrinsic to this [3]catenane. Investigation confirmed no systematic error, and their omission does not significantly affect the reliability or chemical interpretation of the structure.

5 References

1 Chan, T. R., Hilgraf, R., Sharpless, K. B. & Fokin, V. V. Polytriazoles as Copper(I)-Stabilizing Ligands in Catalysis. *Org. Lett.* **6**, 2853–2855 (2004).

2 Lin, Y.-H., Lai, C.-C. & Chiu, S.-H. Five additional macrocycles that allow Na+ ion-templated threading of guest units featuring a single urea or amide functionality. *Org. Biomol. Chem.* **12**, 2907–2917 (2014).

3 Chen, C.-Y. *et al.* Complementarity of 2,6-Dimethanolpyridine and Di(ethylene glycol) in the Complexation of Na+ Ions: Attaching Multiple Copies of [2]Catenane Branches to Isophthalaldehyde-Containing Cores. *J. Org. Chem.* **86**, 13491–13502 (2021).

4 Tse, Y. C., Docker, A., Zhang, Z. & Beer, P. D. Lithium halide ion-pair recognition with halogen bonding and chalcogen bonding heteroditopic macrocycles. *Chem. Commun.* **57**, 4950–4953 (2021).

5 Ng, K.-Y., Felix, V., Santos, S. M., Rees, N. H. & Beer, P. D. Anion induced and inhibited circumrotation of a [2]catenane. *Chem. Commun.*, 1281–1283 (2008).

6 Cosier, J. & Glazer, A. M. A nitrogen-gas-stream cryostat for general X-ray diffraction studies. *Journal of Applied Crystallography* **19**, 105–107 (1986).

7 Agilent (2014). CrysAlis PRO. Agilent Technologies Ltd, Y., Oxfordshire, England.

8 Sheldrick, G. M. SHELXT - Integrated space-group and crystal-structure determination. *Acta Crystallogr., Sect. A* **71**, 3–8 (2015).

9 Sheldrick, G. M. *Acta Crystallogr., Sect. A* **64**, 112–122 (2008).

10 Dolomanov, O. V., Bourhis, L. J., Gildea, R. J., Howard, J. A. K. & Puschmann, H. OLEX2: a complete structure solution, refinement and analysis program. *J. Appl. Cryst.* **42**, 339–341 (2009).
